# Effects of Conjugated Linoleic Acid, Fish Oil and Soybean Oil on PPARs (α & γ) mRNA Expression in Broiler Chickens and Their Relation to Body Fat Deposits

**DOI:** 10.3390/ijms12128581

**Published:** 2011-11-29

**Authors:** Maryam Royan, Goh Yong Meng, Fauziah Othman, Awis Qurni Sazili, Bahman Navidshad

**Affiliations:** 1 Faculty of Veterinary Medicine, University Putra Malaysia, 43400 UPM Serdang, Selangor, Malaysia; E-Mail: ymgoh@vet.upm.edu.my; 2 Institute of Tropical Agriculture, University Putra Malaysia, 43400 UPM Serdang, Selangor, Malaysia; 3 Department of Human Anatomy, Faculty of Medicine and Health Sciences, University Putra Malaysia, 43400 UPM Serdang, Selangor, Malaysia; E-Mail: fauziah@medic.upm.edu.my; 4 Department of Animal Science, University Putra Malaysia, 43400 UPM Serdang, Selangor, Malaysia; E-Mail: awis@putra.upm.edu.my; 5 Department of Animal Science, University of Mohaghegh Ardabili, P.O. Box: 179, Ardabil, Iran; E-Mail: bnavidshad@uma.ac.ir

**Keywords:** CLA, PUFA, PPARs, broiler chickens

## Abstract

An experiment was conducted on broiler chickens to study the effects of different dietary fats (Conjugated linoleic acid (CLA), fish oil, soybean oil, or their mixtures, as well as palm oil, as a more saturated fat), with a as fed dose of 7% for single fat and 3.5 + 3.5% for the mixtures, on Peroxisome Proliferator-Activated Receptors (PPARs) gene expression and its relation with body fat deposits. The CLA used in this experiment was CLA LUTA60 which contained 60% CLA, so 7% and 3.5% dietary inclusions of CLA LUTA60 were equal to 4.2% and 2.1% CLA, respectively. Higher abdominal fat pad was found in broiler chickens fed with a diet containing palm oil compared to chickens in the other experimental groups (*P* ≤ 0.05). The diets containing CLA resulted in an increased fat deposition in the liver of broiler chickens (*P* ≤ 0.05). The only exception was related to the birds fed with diets containing palm oil or fish oil + soybean oil, where contents of liver fat were compared to the CLA + fish oil treatment. PPARγ gene in adipose tissue of chickens fed with palm oil diet was up-regulated compared to other treatments (P ≤ 0.001), whereas no significant differences were found in adipose PPARγ gene expression between chickens fed with diets containing CLA, fish oil, soybean oil or the mixture of these fats. On the other hand, the PPARα gene expression in liver tissue was up-regulated in response to the dietary fish oil inclusion and the differences were also significant for both fish oil and CLA + fish oil diets compared to the diets with palm oil, soybean oil or CLA as the only oil source (*P* ≤ 0.001). In conclusion, the results of present study showed that there was a relationship between the adipose PPARγ gene up-regulation and abdominal fat pad deposition for birds fed with palm oil diet, while no deference was detected in n-3 and n-6 fatty acids, as well as CLA on PPARγ down regulation in comparison to a more saturated fat. When used on its own, fish oil was found to be a more effective fat in up-regulating hepatic PPARα gene expression and this effect was related to a less fat deposition in liver tissue. A negative correlation coefficient (−0.3) between PPARα relative gene expression and liver tissue fat content confirm the anti-lipogenic effect of PPARα, however, the change in these parameters was not completely parallel.

## 1. Introduction

Broiler chickens have a high ability for lipid biosynthesis. It is reported that total body lipids of growing chickens doubles every 5.5 days, and they reach to the maximum rate of hepatic fatty acid synthesis at 7 weeks of age [1,2].

Liver is the main site of lipogenesis in birds and based on equal weight, this is 20 times more than adipose tissue lipogenesis capacity [3]. In contrast, adipose tissue is the major site of lipid synthesis in pigs, ruminants and laboratory rodents [4]. It has been shown that neglectable lipogenesis occurs in chicken adipose tissue [5].

Previous reports suggest that in both birds and mammals, PUFAs reduce lipid synthesis [6–10] and increase fatty acid oxidation [10–12] and diet-mediated thermogenesis [13], while saturated and monounsaturated fatty acids have no inhibitory effects on body fat deposition [8].

Fish oil as a well-known source of long chain n-3 polyunsaturated fatty acids, eicosapentaenoic acid (EPA 20:3 *n*-3) and docosahexaenoic acid (DHA 22:5 *n*-3) have been reported to reduce the activity of enzymes involved in hepatic triglyceride synthesis, so that EPA (20:5*n*-3) reduced the activity of acyl-CoA;1,2-diacylglycerol O-acyltransferase which catalyses the final step of triglyceride synthesis [14].

Conjugated linoleic acid (CLA) is a general name for the positional and geometric isomers of linoleic acid (9cis, 12cis octadecadienoic acid; 18:2n 6). It has reported that dietary CLA decreases fat deposition in several mammalian species and chickens [15,16]. It is suggested CLA provoke fatty acid β-oxidation in skeletal muscle [17,18]. This effect is shown by an up-regulation of carnitine palmitoyl transferase I (CPT-I, the main enzyme for β-oxidation) in skeletal muscles [19–22].

The n-6 PUFA subtypes and specially linoleic acid (18:2 *n*-6), which is plentifully found in soybean oil, also inhibit the activity of enzymes associated with hepatic lipogenesis. Allmann and Gibson [23] first reported that within two days of inclusion just 2% linoleic acid to a high-carbohydrate, fat-free diet of mice, the rate of liver fatty acid synthesis and the activities of FAS, glucosc-6- phosphate dehydrogcnase and malic enzyme were reduced by 70%.

In contrast, dietary palmitic (16:0) or oleic (18:1*n*-9) acids which are the dominant fatty acids in palm oil, did not change hepatic fatty acid synthesis [24]. As a matter of fact, it seems that the greater the degree of unstauration of the fatty acid the more fatty acid synthesis is inhibited; DHA (22:6*n*-3) is more potent than EPA (20:5:*n*-3) or arachidonic (20:4*n*-6) [25].

The majority of the metabolic effects of consumed fatty acids are mediated through alterations in gene expression, either directly or indirectly via nuclear hormone receptor such as Peroxisome Proliferator-Activated Receptors (PPARs) [26,27]. PUFAs and CLA isomers acts as ligands for PPARs [28,29].

PPARs are members of the nuclear hormone receptor superfamily [30]. PPARs as the “lipid sensors,” are triggered by fatty acids, a range of fatty acid derivatives and another peroxisome proliferators, like hypolipidemic drugs of the fibrate class to control the expression of physiologically multi- functional genes [31,32]. The most role of Peroxisome Proliferator-Activated Receptor γ (PPARγ) is in initiating adipocyte differentiation and increasing fat deposits [33,34]. Peroxisome Proliferator-Activated Receptor α (PPARα) increase fatty acid (FA) oxidation through up-regulating the expression of the carnitine palmitoyltransferase enzymes and acyl-coenzyme A (CoA) oxidase (ACO) [35,36].

It seems that various isoforms of PPAR have special impact in lipid metabolism, and n-3 and n-6 fatty acids stimulate these PPAR isoforms with different potency. Therefore, PPARα is more potent in activation of peroxisomal enzymes, which are more activated by n-3 than n-6 fatty acids [37].

Most reports for CLA and PUFAs effects on PPARs are based on the experiments carried out using rodents or rodent-derived cells or in-vitro studies. The aim of this study was to investigate the manner that dietary Fish oil (FO, *n*-3 rich), Soybean oil (SO, *n*-6 rich) PUFAs, CLA or more saturated fats (Palm oil, PO) influence PPARs (α and γ) mRNA expression in liver and adipose tissue of broiler chickens.

## 2. Results and Discussion

### 2.1. Liver Fat Content and Abdominal Fat Pad Deposition

Table 1 shows abdominal fat pad deposition and lipid content of liver tissue of birds fed with different dietary fats. Dietary palm oil significantly increased abdominal fat pad deposition (*P* ≤ 0.05), but no differences were found in this respect between other experimental groups.

The CLA-containing diets resulted in increased fat deposition in the liver of broiler chickens (*P* ≤ 0.05). The only exceptions were related to the birds fed PO or FO + SO diets; their liver fat content was comparable to the CLA + FO treatment. The FO resulted in the lowest liver fat content (*P* ≤ 0.05).

### 2.2. PPARs Gene Expression

**Figure 1:**
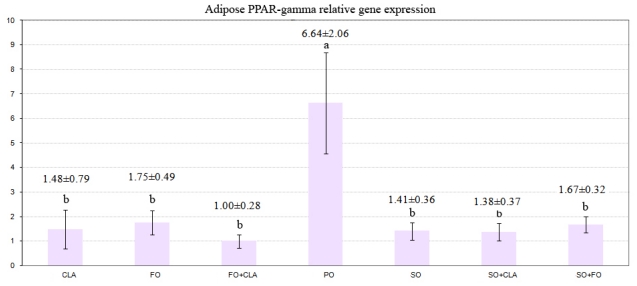
Effect of different dietary fats on Peroxisome Proliferator-Activated Receptor γ (PPARγ) mRNA relative expression in adipose tissue. PO = diet containing Palm oil, SO = diet containing 7% Soybean oil, FO = diet containing 7% Fish oil, CLA = diet containing 4.2% CLA, CLA + SO = diet containing 2.1% CLA + 3.5% Soybean oil, CLA + FO = diet containing 2.1% CLA + 3.5% Fish oil, FO + SO = diet containing 3.5% Fish oil + 3.5% Soybean oil.

Figure 1 shows that the adipose PPARγ gene expression in the palm oil treatment was significantly up-regulated compared with other dietary treatments (*P* ≤ 0.001), whereas no significant differences were observed in the adipose PPARγ gene expression between the treatments containing CLA, fish oil, soybean oil or the mixture of these fats.

**Figure 2:**
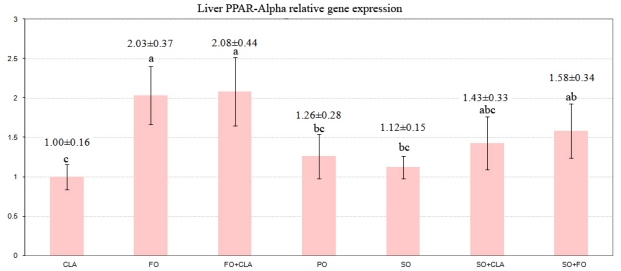
Effect of different dietary fats on PPARα mRNA relative expression in liver tissue. PO = diet containing Palm oil, SO = diet containing 7% Soybean oil, FO = diet containing 7% Fish oil, CLA = diet containing 4.2% CLA, CLA + SO = diet containing 2.1% CLA + 3.5% Soybean oil, CLA + FO = diet containing 2.1% CLA + 3.5% Fish oil, FO + SO = diet containing 3.5% Fish oil + 3.5% Soybean oil.

In contrast, the PPARα gene expression in the liver tissue (Figure 2) was up-regulated in response to the dietary fish oil or fish oil + CLA inclusions and the difference was rather significant compared to the PO, SO and CLA treatments (*P* ≤ 0.001).

Some previous reports have demonstrated that dietary n-3 and n-6 PUFAs and also CLA isomers inhibit lipogenesis, while saturated and monounsaturated fatty acids have no inhibitory effects [8,16]. In the present study, the higher expression of PPARγ gene in the adipose tissue of birds fed with palm oil compared to the other treatment indicated a suppression effect by PUFA from soybean oil and fish oil and also CLA, while the source of PUFAs seemed not to influence the PPARγ expression. This finding is confirmed by the higher abdominal fat pad deposited in birds fed palm oil in comparison to other treatments.

It another report, the abdominal fat pad weights in 5-wk-old broiler chickens orally administered troglitazone, a synthetic PPARγ ligand, were significantly increased due to stimulation of PPARγ activity and increases in lipoprotein lipase (LPL) mRNA levels in abdominal adipose tissues [38]. It seems that PPARγ is involved in the regulation of both the hyperplasia of adipocytes and hypertrophic growth in broiler chickens and that the up-regulation of PPARγ in the hyperplasia step of adipose tissue markedly modulates fatness in broiler chickens [39].

In mammals, liver takes up glycerol and converts it into glucose, but adipose tissue takes up glucose and converts it into glycerol 3-phosphate, which becomes included into triglycerides. It is well-known that the uptake of glucose into adipocytes and its conversion to triglycerides is motivated by PPARγ [40,41].

Nevertheless, the PPARγ expression in the liver of the experimental birds was not examined in this study because the expression of PPARγ in the liver of birds was shown to be negligible in previous research [42]. The reasons for this particular variation maybe the fundamental differences between mammals and birds in the role of liver in fat metabolism. In chickens, liver is the most important lipogenetic organ and adipose tissue only acts as storage tissue [43]; it is important to note that the lipogenic activity in the liver is considerably more than in the adipose tissue of avian [44,45]. The effect of PUFA on PPARs is a function of the ligand affinity of PPARs. The present study revealed that the diet containing palm oil supplied 4.7 and 1.7 times more C16:0 and C18:0 fatty acids respectively, compared to the mean of the other treatments. Generally, PPARs have a binding affinity order: PUFA > MUFA > SFA [46,47]. Kersten [48] reported that the highest binding affinity was found for α-linoleic acid, *i.e*., an intermediate in the synthesis of arachidonic acid from linoleic acid.

In order to formulate isocaloric diets in the present research, the diet containing palm oil had inevitably higher fat content as compared to other experimental diets. There are many reports which have similarly indicated that PPARγ levels are affected by dietary fat content as well [38]. Feeding mice with high dietary fat increased the expression of PPARγ in adipose tissue [49]. It has been reported that exposure of human subcutaneous adipose tissue to a triglyceride mixture for 5 hours markedly increased the PPARγ mRNA expression [50]. Previous reports have recognized that AQP7 and probably glycerol kinase are the direct PPARγ target genes in adipose tissue [51]. Zhao *et al*. [52] proved that PPARγ is involved in direct upregulation of cGPDH gene in adipocytes. PPAR γ is likely to regulate many other genes in adipose tissue, although the only other known case is phosphoenolpyruvate carboxykinase which catalyses glycerol synthesis. PPARγ not only is an important modulator for adipose tissue maturation but is also a key coordinator of fat uptake and storage by controlling genes such as fatty acid binding proteins and lipoprotein lipase [53].

Feeding fish oil or the mixture of CLA + fish oil to broiler chickens has been found to cause a significant up-regulation of PPARα gene compared to feeding CLA, soybean oil or palm oil. This finding suggests that n-3 PUFA present in fish oil is capable of binding to and activating PPARα. This finding is in agreement with some studies which used rodents as their sample and whereby the administration of fish oil was observed to cause a stronger up-regulation of PPARα and its target genes [54]. The observation for broiler chickens in the present study is similar to that seen in turkeys [55], mice [56], and humans [57], indicating that a considerable capacity for peroxisomal FA β-oxidation in liver is available in broiler chickens treated with n-3 PUFA. Some previous reports [58,59] have suggested that a less fat deposition in the liver of birds fed fish oil is due to the activated β-oxidation pathway in hepatocytes, which causes the conversion of TG into glycerol and fatty acids, and also improves the hepatic uptake of the released NEFA.

In the present study, however, it seemed that there was no exact relationship between PPARα gene expression and fat deposition in hepatocytes, since the significant up-regulation of hepatic PPARα gene in birds fed with FO + CLA diet did not lead to a reduced fat deposition in liver. Hence, this observation suggests that the effect of CLA on increasing liver fat deposition is possibly independent of the PPARα gene expression.

In addition, the data gathered in the present study also showed that the CLA mixture administered to the chickens, with cis-9, trans-11 and trans-10, cis-12 CLA as the main isomers, did not significantly up-regulate the gene expression of PPARα, although there was a numerical increase in the mRNA level of PPARα for the CLA + FO treatment. The disagreement between the findings gathered in the present study and that of the study on rat liver cells is probably because the rat liver cells have a very high gene expression of PPARα in the liver, which is several fold greater than those in other species [60,61]. The finding indicating that the CLA increases hepatic TAG concentration agrees with recent studies conducted on laying hens [62]. At the same time, it could be noted that trans-10, cis-12 CLA isomer, consisting of about 50% of the total dietary CLA isomers, caused similar effects in the liver of chickens. Interestingly, no decrease was observed in the abdominal fat pad weight in the chickens fed with the diet containing fish oil compared to the CLA, and soybean oil treatments in this study.

## 3. Experimental Section

Day old broiler chicks (Ross 308) were fed a commercial starter diet for their first 10 days and then allocated to the experimental grower (11–28 d) and finisher (29–42 d) diets. Chicks were placed randomly into each of 28 litter pens (20 chicks per 1.5 × 1.5 m^2^ pen). A lighting program of 23L:1D was used for the entire 42-d raring period. The birds were housed in an environmentally-controlled room (temperature gradually decreased from 32 °C on their first day to 18 °C at 42 days of age with about 60% relative humidity), and they had free access to feed (mash) and water.

Seven isocaloric and isonitrogenous diets were formulated containing: 7% Soybean oil (SO), 3.5% LUTA-CLA 60 (CLA), 7% Fish oil (FO), 3.5% LUTA-CLA 60 + 3.5% Soybean oil (CLA + SO), 3.5% Fish oil + 3.5% Soybean oil (FO + SO), 3.5% LUTA-CLA 60 + 3.5% Fish oil (CLA + FO) or up to 12.9% Palm oil (PO), as-fed basis.

The CLA supplement used in this study (LUTA-CLA 60) prepared and supplied by BASF Company (Germany) and contained 30% Isomer 9c, 11t and 30% isomer 10t, 12c of conjugated linoleic acid plus mostly oleic acid, then 7% and 3.5% dietary inclusion of CLA was equal to 4.2% and 2.1% respectively. The higher fat inclusion in palm oil diet was because of the lower metabolisable energy of palm oil. The birds were fed a grower diet until 24 days of age followed by a finishing diet at 25 to 42 days of age. The experimental diets were formulated using Ross Co (2002) guideline with, 3175–3205 and 3225–3241 Kcal/kg metabolisable energy and 21 and 19 percent crude protein for grower and finisher phases, respectively.

Tables 2 and 3 show the chemical composition and fatty acid analysis of the experimental diets. At the end of experiment period (42d), eight male chicks per treatment were weighted individually and slaughtered. Abdominal fat pad (including fat surrounding gizzard, bursa of fabricius, cloaca and adjacent muscles) was removed and weighed individually. The percentage of abdominal fat weight was expressed as a ratio of body weight. Samples of liver were stored at −20 °C until lipid content analysis. Additional samples from liver and abdominal fat pad tissues were collected, flash-frozen in liquid nitrogen and stored at −80 °C for RNA extraction.

Lipid content of liver tissue was measured by the method of Folch *et al*. [63]. Total RNA of each tissue sample was extracted by Quiagen, RNeasy Lipid Tissue Mini Kit according to manufacturer’s instruction. The integrity and purity of RNA were tested by measurement of optical density (ratios at 260 and 280 nm being greater than 1.9) and by electrophoresis using ethidium bromide staining.

For each analyzed tissue, 5 μg of RNA was reverse-transcribed by using the QuantiTect Rev. Transcription Kit according to the manufacturer’s protocol. Reverse transcription was carried out at 42 °C for 15 min. The reverse transcriptase enzyme was heat-treated at 95 °C for 3 min. A master mix of 20 μL containing 0.5 μL diluted RNA, 10 μL SYBR Green I PCR Master Mix, 0.5 μL forward primer (5 pM/μL), 0.5 μL reverse primer (5 pM/μL), and 8.5 μL water was prepared to perform real-time RT-PCR. The forward and reverse primers for PPARs and β-Actin cDNA were derived from previous reports (Table 4). Each real-time RT-PCR assay was run along with a dilution series of the standard that served as calibrator. A no-template control was also included with each run.

All the real-time PCR runs were conducted in a final volume of 20 μL of QuantiTect SYBR Green PCR Kits (Qiagen) containing DNA intercalated dye SYBR Green 1 dye. Real time quantification was monitored by determining the raise in fluorescence because of the joining of SYBR Green dye to double-stranded DNA at the end of each amplification cycle. At the end of the PCR amplification, dissociation was carried out through slowly heating the PCR products from 55 to 95 °C and continuous recording of the reduction in SYBR Green fluorescence following the dissociation of double-stranded DNA. The cycle at which an increase in fluorescence more than a definite baseline can be first detected was described as threshold cycle (Ct), and was determined for each sample.

The mRNA concentrations were measured on the basis of PCR efficiency and Ct deviation of an unknown sample against a control according to 2^−ΔΔCT^ method [66]. The β-Actin gene was chosen as a reference gene. For validation of the method, cDNA was synthesized from 10-fold serially diluted RNA samples and amplified by real time PCR using target gene-specific primers. Each PCR run included a no-template control and replicates of control and unknown samples. Runs were performed in duplicate. The treatment with the lowest mRNA expression was chosen as calibrator treatment. Based on the 2^−ΔΔCT^ method, the output data were obtained as the fold change in target gene expression normalized to the β-Actin gene (endogenous control) and relative to the calibrator treatment.

### Statistical Analyses

For abdominal fat pad percentage and tissue fat contents, data sets of completely randomized design with seven treatments and eight replicates, were compared across the treatments using the one-way analysis of variance (ANOVA) procedure. Significant means were then elucidated using the Duncan multiple range tests. All statistical tests were conducted at 95% confidence level using the SAS program (SAS, 9.1) [67].

The comparison of gene expression data was carried out using Microsoft Excel® macro genex version 1.1, Bio-Rad (2004) and the significance of difference was determined using REST 2009 software which does the mutual comparisons following data simulation. A correlation coefficient between PPARα relative gene expression and liver tissue fat content was calculated using Microsoft Office Excel.

## 4. Conclusions

The results of the present study showed that there was a relationship between the adipose PPARγ gene up-regulation and abdominal fat pad deposition in the broiler chickens for the PO treatment, whereas no difference was observed for the n-3 and n-6 fatty acids or the CLA on the PPARγ gene expression. Therefore, in combination with CLA, fish oil is the most effective fat to be used in upregulating PPARα, and this effect is related with a less fat deposition in liver. A negative correlation coefficient (−0.3) between PPARα relative gene expression and liver tissue fat content confirms the anti-lipogenic effect of PPARα. The birds fed more dietary CLA showed the highest liver fat content and the lowest PPARα gene expression. However, the changes in PPARα gene expression and liver fat content in the overall treatments were not completely parallel.

## Figures and Tables

**Table 1:** Liver Fat content and abdominal fat pad of the experimental birds.

	Liver Fat content (% of dry matter)	Abdominal Fat Pad (% of live weight)
PO 1	3.34 b	2.35 a
SO	2.85 c	1.86 b
FO	2.42 d	2.03 b
CLA	4.22 a	2.14 b
CLA + SO	4.13 a	2.13 b
CLA + FO	3.86 a,b	2.14 b
FO + SO	3.16 b,c	2.12 b
Pooled SEM	0.16	0.08

a–d Means with different superscripts within column differ significantly at *P* < 0.05.

1 CLA used in this experiment was CLA LUTA60 which contains 60% CLA, then 7% and 3.5% dietary inclusion of CLA will be equal to 4.2% and 2.1% respectively. 2PO= diet containing Palm oil, SO = diet containing 7% Soybean oil, FO = diet containing 7% Fish oil, CLA = diet containing 4.2% CLA, CLA + SO = diet containing 2.1% CLA + 3.5% Soybean oil, CLA + FO = diet containing 2.1% CLA + 3.5% Fish oil, FO + SO = diet containing 3.5% Fish oil + 3.5% Soybean oil.

**Table 2:** Ingredients and chemical compositions of the experimental diets.

Ingredients (as-fed basis)	Starter (1-10 d)	Grower (11–28 days of age)							Finisher (29–42 days of age)						
	
		PO^2^	SO	FO	CLA	FO + SO	CLA + SO	CLA + FO	PO	SO	FO	CLA	FO + SO	CLA + SO	CLA + FO
Corn (%)	60.23	48.46	53.99	53.99	55.8	53.99	54.13	54.00	52.62	57.98	57.98	59.50	57.98	59.06	58.92
Soy meal (%)	30.81	30.56	32.27	32.27	28.6	32.27	31.96	32.26	27.76	30.27	30.27	26.38	30.27	28.30	28.60
Fish Meal (%)	5.37	5.00	3.00	3.00	5.00	3.00	3.20	3.01	3.00	1.00	1.00	2.99	1.00	1.70	1.51
Soybean oil (%)	-	-	7.00	-	-	3.50	3.50	-	-	7.00	-	-	3.50	3.50	-
Fish oil	-	-	-	7.00	-	3.50	-	3.50	-	-	7.00	-	3.50	-	3.50
Palm oil (%)	-	12.60	-	-	-	-	-	-	12.92	-	-	-	-	-	-
CLA (%) 1	-	-	-	-	7.00	-	3.50	3.50	-	-	-	7.40	-	3.50	3.50
Oyster shell (%)	1.41	1.34	1.42	1.42	1.33	1.42	1.41	1.42	1.30	1.39	1.39	1.30	1.39	1.35	1.36
DCP (%)	0.51	0.48	0.66	0.66	0.52	0.66	0.64	0.66	0.69	0.84	0.84	0.71	0.84	0.81	0.82
Salt (%)	0.25	0.29	0.32	0.32	0.28	0.32	0.31	0.32	0.32	0.35	0.35	0.32	0.35	0.34	0.34
Vit-Min P (%) 3	1.00	1.00	1.00	1.00	1.00	1.00	1.00	1.00	1.00	1.00	1.00	1.00	1.00	1.00	1.00
DL-Met (%)	0.26	0.23	0.25	0.25	0.23	0.25	0.25	0.25	0.17	0.18	0.18	0.16	0.18	0.18	0.18
L-Lys (%)	0.15	0.04	0.09	0.09	0.24	0.09	0.09	0.09	0.22	-	-	0.25	-	0.27	0.27
Analysis															
ME (Kcal/Kg)	2860	3175	3205	3211	3175	3208	3175	3175	3225	3235	3241	3225	3238	3225	3225
CP (%)	22.5	21.00	21.00	21.00	21.00	21.00	21.00	21.00	19.00	19.00	19.00	19.00	19.00	19.00	19.00
Crude Fat (%)	2.86	15.00	9.52	9.52	9.65	9.52	9.54	9.52	15.34	9.55	9.55	10.07	9.50	9.61	9.60
Linoleic a (%)	1.46	2.63	5.37	1.39	5.27	3.38	5.30	3.31	2.74	5.45	1.46	5.56	3.46	5.40	3.40
Ca (%)	0.95	0.90	0.90	0.90	0.90	0.90	0.90	0.90	0.85	0.85	0.85	0.85	0.85	0.85	0.85
Ava P (%)	0.48	0.45	0.45	0.45	0.45	0.45	0.45	0.45	0.425	0.425	0.425	0.43	0.43	0.43	0.43
Na (%)	0.15	0.16	0.16	0.16	0.16	0.16	0.16	0.16	0.16	0.16	0.16	0.16	0.16	0.16	0.16
Lys (%)	1.37	1.23	1.23	1.23	1.34	1.23	1.23	1.23	1.20	1.02	1.02	1.20	1.01	1.20	1.20
Met (%)	0.66	0.60	0.60	0.60	0.60	0.60	0.60	0.60	0.48	0.48	0.48	0.48	0.48	0.49	0.49
Met + Cys (%)	1.04	0.95	0.95	0.95	0.95	0.95	0.95	0.95	0.80	0.80	0.80	0.80	0.80	0.80	0.80

1 CLA used in this experiment was CLA LUTA 60 which contains 60% CLA, then 7% and 3.5% dietary inclusion of CLA will be equal to 4.2% and 2.1% respectively;

2 PO = diet containing Palm oil, SO = diet containing 7% Soybean oil, FO = diet containing 7% Fish oil, CLA = diet containing 4.2% CLA, CLA + SO = diet containing 2.1% CLA + 3.5% Soybean oil, CLA + FO = diet containing 2.1% CLA + 3.5% Fish oil, FO + SO = diet containing 3.5% Fish oil + 3.5% Soybean oil.

3 Mineral premix provided per kg of ration with 50 mg Fe, 70 mg Mn, 50 mg Zn, 7 mg Cu, 0.4 mg Co, 0.17 mg Se, and 0.75 mg I. Vitamin premix provided per kg of ration with 6,000,000 IU vitamin A, 1.500,000 IU vitamin D3, 15,000 IU vitamin E, 2.5 mg vitamin K3, 0.02 mg vitamin B12, 3,000 mg riboflavin, 7000 mg pantothenic.

**Table 3:** Fatty acid composition (g/kg diet) of experimental diets.

	Grower							Finisher						
		
	PO 1	SO	FO	CLA 2	FO + SO	CLA + SO	CLA + FO	PO	SO	FO	CLA	FO + SO	CLA + SO	CLA + FO
C14:0	1.52	0.24	3.211	0.33	1.65	0.23	1.72	1.52	0.12	3.13	0.22	1.62	0.11	1.62
C16:0	58.63	10.71	20.4	8.43	15.52	9.32	14.10	59.51	10.16	19.91	8.12	15.06	8.92	13.81
C16:1	0.86	0.66	4.50	0.38	2.51	0.37	2.42	0.84	0.32	4.31	0.22	2.36	0.18	3.00
C17:0	0.13	0.13	1.03	0.08	0.46	0.11	0.45	0.12	0.02	0.91	0.13	0.47	0.03	0.52
C17:1	0.12	0.04	0.53	0.02	0.28	0.04	0.31	0.04	0.01	0.52	0.01	0.27	0.02	0.21
C18:0	6.12	3.50	4.27	3.71	3.89	3.53	3.92	6.12	3.40	4.17	3.74	3.82	3.42	3.84
C18:1c	54.33	23.72	31.42	24.24	27.59	23.61	27.31	55.00	23.22	30.86	24.47	27.12	23.31	27.12
C18:2c	23.91	49.41	15.84	15.43	32.62	32.32	15.48	24.88	50.0-6	16.50	16.08	33.26	33.14	16.3
C18:3 alpha	0.02	0.02	0.12	0.03	0.07	0.02	0.07	0.02	0.02	0.11	0.03	0.07	0.02	0.07
C18:3 gamma	0.31	0.34	1.11	0.33	0.73	0.27	0.71	0.21	0.20	1.07	0.32	0.61	0.34	0.74
C20:0	0.02	0.03	0.12	0.04	0.11	0.03	0.12	0.02	0.04	0.14	0.02	0.09	0.01	0.11
CLA-9c, 11t	0.05	0.00	0.00	21.00	0.00	10.49	10.51	0.00	0.00	0.00	22.20	0.00	10.50	10.50
CLA-10c,12t	0.04	0.00	0.00	21.00	0.00	10.52	10.46	0.00	0.00	0.00	22.20	0.00	10.50	10.50
C20:5 n-3	0.44	0.18	4.11	0.44	2.23	0.20	2.22	0.23	0.13	4.03	0.21	2.00	0.08	2.06
C22:6 n-3	0.92	0.51	9.76	0.92	5.07	0.61	5.20	0.46	0.16	9.50	0.54	4.82	0.32	4.92
n6/n3	18.31	69.00	1.23	11.82	4.54	39.84	2.18	34.92	157.22	1.33	22.54	4.91	79.48	2.40

1 PO = diet containing Palm oil, SO = diet containing 7% Soybean oil, FO = diet containing 7% Fish oil, CLA = diet containing 4.2% CLA, CLA + SO = diet containing 2.1% CLA + 3.5% Soybean oil, CLA + FO = diet containing 2.1% CLA + 3.5% Fish oil, FO + SO = diet containing 3.5% Fish oil + 3.5% Soybean oil;

2 CLA used in this experiment was CLA LUTA60 which contains 60% CLA, then 7% and 3.5% dietary inclusion of CLA will be equal to 4.2% and 2.1% respectively.

**Table 4:** The forward and reverse primers for PPARα, PPARγ and β-actin.

Gene	Primer	Base pairs	Annealing temperature(Ċ)	References
B-actin	F-5′ATGAAGCCCAGAGCAAAAGA3′	223	62	[64]
	R-5′GGGGTGTTGAAGGTCTCAAA3′			
PPARα	F-5′AGGCCAAGTTGAAAGCAGAA3′	217	60	[64]
	R-5′GTCTTCTCTGCCATGCACAA3′			
PPARγ	F-5′GACCTTAATTGTCGCATCCAT3′	237	61	[65]
	R-5′CGGGAAGGACTTTATGTATGA3′			
